# Admission of Air into the Veins

**Published:** 1833

**Authors:** 


Art. VI.—*Admission of Air into the Veins.*—We do not recollect to have published any thing on this subject. The following case, extracted from the Boston Medical and Surgical Journal, may not be uninteresting to our surgical readers.

A mantuamaker, aged 18, of a feeble constitution, bore for many years past a scrofulous tumor at the superior and lateral part of the neck, which increased gradually every year. It was about the size of the head of a fœtus *a terme*, when the patient entered the hospital La Charite, the 27th Sept. 1832.

M. Roux decided to extirpate it. He made a crucial incision of the integuments, dissected up the flaps, dividing the cellular adhesions which united the tumor to the surrounding parts, and tying in his course some small arteries that threw out the blood. He then raised the tumor with the left hand, to continue the dissection, when suddenly a peculiar sound was heard, a kind of "sufflement," analogous to the sound made by air when a small quantity of it (quelques bulles) enters the vacuum of an air pump. At the same moment the patient uttered a plaintive cry, throwing herself from one side of the bed to the other; the inspirations became long and painful; the respiratory muscles were seen to contract with force; the pulsations of the heart increased, the arterial undulations becoming more feeble. A rale was heard, produced by the passage of the air through the mucosities accumulated in the bronchiæ; the respiration became more and more heaving. Finally there supervened one long inspiration, followed by a short one, and all the apparent symptoms of death.

On the appearance of this accident, M. Roux suspected the introduction of air into the veins, compressed the wound with the fingers, caused frictions to be made over the precordial region, and glasses of cold water to be thrown upon the face; at the same time the nostrils were excited with the feather of a pen dipped in ammonia. After some minutes the heart was perceived to recommence its pulsations, and the respiration returned. The patient being questioned, was at first only able to stammer, and to express herself by interrupted sounds, similar to those made by patients who are affected with a paralysis of the vocal organs. A certain quantity of mucus now escaped from the mouth. Finally she commenced to articulate and to complain of her trouble.

M. Roux discontinued the operation, tied the vessels, and strangled with a double ligature the tumor, three quarters dissected. The patient was covered with very hot cloths, and she had given her alternately, every hour, a spoonful of ether and of Malaga wine. Until the 5th day, no accident. On the 6th, the tumor, reduced to a putrid state, was taken away without difficulty. Suddenly, on the morning of the 7th day, she was seized with slight oppression, speech embarrassed, succeeded by a comatose state, and death during the night.

*Autopsy.*—The wound was bounded below by the clavicle, above by the mastoid region, within by the larynx and the muscles of the trachea, on the outside by the posterior muscles of the neck. The tumor reposed immediately on the cellular sheath of the carotid arteries and jugular vein. It was covered by the skin and the sterno-mastoid muscle. Skin generally pale; the cellular sheath of the vessels of the neck was divided; the internal jugular vein was divided transversely, the lower mouth of the vessel being perceived gaping, with its parietes already thickened. A probe being introduced, penetrated as far as the subclavian vein. The carotid artery and pneumo-gastric nerve were untouched.

The lungs were crepitant; bronchial ramifications of the right side engorged and filled with a spumous serosity; left lung less engorged; emphysematous points were perceived under the poumonic pleura. Cavities of the heart empty. The descending aorta, being punctured in different places, permitted a perceptible quantity of air bubbles to escape, mixed with a bloody serum; the iliac arteries presented the same phenomenon, but to a less marked degree. The arteries at the base of the brain did not contain any air. The cerebral ventricles enclosed a small quantity of yellow serosity. The veins offered nothing anormal. The digestive apparatus healthy.

